# Supplementary material for: Lytic Spectra of Tailed Bacteriophages: A Systematic Review and Meta-Analysis
Source: Viruses. 2024 Dec 4;16(12):1879. doi: 10.3390/v16121879 (PMC11680127; doi:10.3390/v16121879)
Supplement: Supplementary file 1 [file viruses-16-01879-s001.zip › Supplement/Code S1.pdf]

```r
# ----------------------------------------------------------------------------
# ----------------------------------------------------------------------------
# ------------------- Lytic spectra of tailed bacteriophages: -----------------
# -------------------- a systematic review and meta-analysis ------------------
#
# ----------- Ivan M. Pchelin, Andrei V. Smolensky, Daniil V. Azarov -----------
# ------------------------- and Artemiy E. Goncharov -------------------------
#
# ------ Code S1. Annotated R code for data analysis and figure generation -----
# ----------------------------------------------------------------------------
# ----------------------------------------------------------------------------

# The analysis was done in R v4.3.3

# Import libraries
library(corrplot) # v0.94
library(cowplot) # v1.1.3
library(dplyr) # v1.1.4
library(ggplot2) # v3.5.1
library(patchwork) # v1.3.0
library(RColorBrewer) # v1.1-3
library(readxl) # v1.4.3
library(reshape2) # v1.4.4
library(tidyr) # v1.3.1
library(viridis) # v0.6.5, also loads required package viridisLite v0.4.2

# Import data
phagedata <- read_xlsx('Data S1.xlsx', na = 'NA', sheet = 'Data', col_types =
                         c('list', rep('guess', 24)))


################################################################################
############################## Data set overview ###############################
################################################################################

# Host genera
host_genera <- phagedata %>%
  select(Host1genus, Phage_genome) %>%
  distinct(Host1genus, Phage_genome, .keep_all = FALSE) %>%
  group_by(Host1genus) %>%
  mutate(n = n()) %>%
  distinct(Host1genus, n) %>%
  arrange(desc(n)) %>%
  ungroup() %>%
  filter(n >= 5)

908*0.75 # 681 phages constitute 75% sample
host_genera
169 + 109 + 84 + 75 + 71 + 58 + 56 + 42 + 37 # 701 phages in top 9 genera
701/908 # 77% data set taken by top 9 host genera

# Visualise host genera with 5+ phages arranged by prevalence.
# The selection was mentioned in the caption as "top 22 genera are shown"
ggplot(host_genera, aes(reorder(Host1genus, n, decreasing = FALSE),
                        n/908*100)) +
  geom_col(fill = '#668888') +
  coord_flip() +
  xlab('Host') +  ylab('Proportion of all bacteriophages (%)') +
  theme_minimal() +
  theme(axis.title = element_text(size = 8),
        axis.text = element_text(size = 6, colour = 'grey20'),
        axis.text.y = element_text(face = 'italic'),
        axis.line = element_line(colour = 'grey20', linewidth = 0.15),
        axis.ticks = element_line(colour = 'black', linewidth = 0.35),
        panel.grid.major = element_blank(),
        panel.grid.minor = element_blank(),
        panel.background = element_blank(),
        panel.border = element_rect(colour = 'grey20', linewidth = 0.5,
                                    fill = NA),
```

```
      plot.background = element_blank(),
      plot.margin = unit(c(0.25,0.25,0.25,0.25), 'cm'))

ggsave('Figure 2a data set overview.pdf', width = 14, height = 6, units = c('cm'))

# Distribution of genome size coloured by morphology
my_morphological_colours <- c(rgb(1, 184, 170, maxColorValue = 255),
                              rgb(242, 200, 15, maxColorValue = 255),
                              rgb(253, 98, 94, maxColorValue = 255), '#525252')
names(my_morphological_colours) <- c('podovirus', 'myovirus', 'siphovirus',
                                     'not determined')
colScale_morphological <- scale_fill_manual(name = 'Bacteriophage\nmorphology',
                   values = my_morphological_colours)

genomes_and_morphology <- phagedata %>%
  select(Genome_size, Morphology, Phage_genome) %>%
  mutate(Morphology_mod = replace_na(Morphology, 'not determined'))

genomes_and_morphology$Morphology_mod <-
  factor(genomes_and_morphology$Morphology_mod,
         levels = c('siphovirus', 'podovirus', 'myovirus', 'not determined'))


Plot1 <- ggplot(genomes_and_morphology, aes(Genome_size/1000)) +
  geom_histogram(aes(fill = Morphology_mod), bins = 50) +
  xlab('Genome size (kbp)') +  ylab('Number of bacteriophages') +
  theme_minimal() +
  colScale_morphological +
  scale_x_continuous(limits = c(0, 375), expand = c(0.02, 0.02), n.breaks = 20) +
  scale_y_continuous(limits = c(0, 300), expand = c(0.02, 0.03)) +
  theme(axis.title = element_text(size = 8, colour='white'),
      axis.text = element_text(size = 6, colour='white'),
      axis.ticks = element_line(colour = 'white', linewidth = 0.35),
      panel.grid = element_blank(),
      panel.background = element_blank(),
      plot.background = element_blank(),
      legend.position = c(0.87, 0.74),
      legend.background = element_rect(colour = 'grey20', linewidth = 0.15),
      legend.key.size = unit(0.2, 'cm'),
      legend.title = element_text(size = 6, colour = 'grey20'),
      legend.text = element_text(size = 6, colour = 'grey20'),
      plot.margin = unit(c(0.25,0.25,0.25,0.25), 'cm'))

# Prepare bar contours separately
Plot2 <- ggplot(genomes_and_morphology, aes(Genome_size/1000)) +
  geom_histogram(fill = NA, colour = 'black', linewidth = 0.15, bins = 50) +
  xlab('Genome size (kbp)') +  ylab('Number of bacteriophages') +
  theme_minimal() +
  colScale_morphological +
  scale_x_continuous(limits = c(0, 375), expand = c(0.02, 0.02),
                     n.breaks = 20) +
  scale_y_continuous(limits = c(0, 300), expand = c(0.02, 0.03)) +
  theme(axis.title = element_text(size = 8),
      axis.text = element_text(size = 6, colour='grey20'),
      axis.ticks = element_line(colour = 'black', linewidth = 0.35),
      panel.grid = element_blank(),
      panel.background = element_blank(),
      panel.border = element_rect(colour = 'grey20', linewidth = 0.5,
                                  fill = NA),
      plot.background = element_blank(),
      plot.margin = unit(c(0.25,0.25,0.25,0.25), 'cm'))

ggdraw() +
  draw_plot(Plot1, x = 0, y = 0) +
  draw_plot(Plot2, x = 0, y = 0)

ggsave('Figure 2b data set overview.pdf', width = 13.5,
      height = 6, units = c('cm'))
```

```r
################################################################################
########################## Visualise literature bias ###########################
################################################################################

SHR <- phagedata %>%
  select(Spotting_HR, PMID_or_Title)
SHR <- as.data.frame(na.omit(SHR))

PHR <- phagedata %>%
  select(Genome_size, Plaquing_HR, PMID_or_Title)
PHR <- as.data.frame(na.omit(PHR))

SHR <- SHR %>%
  group_by(PMID_or_Title) %>%
  mutate(N_phages_per_article = n()) %>%
  mutate(bins = cut(Spotting_HR, breaks = seq(0, 1, by = 0.05)))

p1 <- ggplot(SHR, aes(bins, group = desc(N_phages_per_article))) +
  geom_bar(aes(fill = N_phages_per_article), stat = 'count', width = 1) +
  scale_fill_gradient2('n phages per\npublication', low = '#440154FF',
                       mid = 'white', high = '#FDE725FF',
                       aesthetics = 'fill', midpoint = 5) +
  theme_light() +
   xlab('Spotting host range') + ylab('') +
   scale_x_discrete(expand = c(0.05, 0.05)) +
   scale_y_continuous(limits = c(0, 72), expand = c(0.02, 0.03)) +
   theme(axis.title = element_text(size = 8, vjust = +3),
         axis.text.x = element_text(size = 4, angle = 35),
         axis.text = element_text(size = 6, colour='white'),
         axis.ticks.x = element_blank(),
         axis.ticks.y = element_line(colour = 'white', linewidth = 0.35),
         panel.grid = element_blank(),
         panel.background = element_blank(),
         plot.background = element_blank(),
         plot.margin = unit(c(0,0,0,0), 'cm'),
         legend.position = c(0.80, 0.74),
         legend.background = element_rect(colour = 'grey20', linewidth = 0.15),
         legend.key.size = unit(0.2, 'cm'),
         legend.title = element_text(size = 6, colour = 'grey20'),
         legend.text = element_text(size = 6, colour = 'grey20'))

# Prepare bar contours separately
p2 <- ggplot(SHR, aes(bins)) +
  geom_bar(fill = NA, colour = 'black', linewidth = 0.15, width = 1) +
  theme_light() +
  xlab('Spotting host range') + ylab('') +
  scale_x_discrete(expand = c(0.05, 0.05)) +
  scale_y_continuous(limits = c(0, 72), expand = c(0.02, 0.03)) +
  theme(axis.title = element_text(size = 8, vjust = +3),
        axis.text.x = element_text(size = 4, angle = 35),
        axis.text = element_text(size = 6, colour='grey20'),
        axis.ticks.x = element_blank(),
        axis.ticks.y = element_line(colour = 'black', linewidth = 0.35),
        panel.grid = element_blank(),
        panel.background = element_blank(),
        panel.border = element_rect(colour = 'grey20',
                                    linewidth = 0.5, fill = NA),
        plot.background = element_blank(),
        plot.margin = unit(c(0,0,0,0), 'cm'))

p3 <- ggdraw() +
  draw_plot(p1, x = 0, y = 0) +
  draw_plot(p2, x = 0, y = 0)

# Histogram of plaquing host range breadth distribution
PHR <- PHR %>%
  group_by(PMID_or_Title) %>%
  mutate(N_phages_per_article = n()) %>%
  mutate(bins = cut(Plaquing_HR, breaks = seq(0, 1, by = 0.05)))
```

```
p4 <- ggplot(PHR, aes(bins, group = desc(N_phages_per_article))) +
  geom_bar(aes(fill = N_phages_per_article), stat = 'count', width = 1) +
  scale_fill_gradient2('n phages per\npublication', low = '#440154FF',
                       mid = 'white', high = '#FDE725FF',
                       aesthetics = 'fill', midpoint = 5) +
  theme_light() +
  xlab('Plaquing host range') + ylab('') +
  scale_x_discrete(expand = c(0.05, 0.05)) +
  scale_y_continuous(limits = c(0, 72), expand = c(0.02, 0.03)) +
  theme(axis.title = element_text(size = 8, vjust = +3),
        axis.text.x = element_text(size = 4, angle = 35),
        axis.text = element_text(size = 6, colour='white'),
        axis.ticks.x = element_blank(),
        axis.ticks.y = element_line(colour = 'white', linewidth = 0.35),
        panel.grid = element_blank(),
        panel.background = element_blank(),
        plot.background = element_blank(),
        plot.margin = unit(c(0,0,0,0), 'cm'),
        legend.position = c(0.80, 0.74),
        legend.background = element_rect(colour = 'grey20', linewidth = 0.15),
        legend.key.size = unit(0.2, 'cm'),
        legend.title = element_text(size = 6, colour = 'grey20'),
        legend.text = element_text(size = 6, colour = 'grey20'))

# Prepare bar contours separately
p5 <- ggplot(PHR, aes(bins)) +
  geom_bar(fill = NA, colour = 'black', linewidth = 0.15, width = 1) +
  theme_light() +
  xlab('Plaquing host range') + ylab('') +
  scale_x_discrete(expand = c(0.05, 0.05)) +
  scale_y_continuous(limits = c(0, 72), expand = c(0.02, 0.03)) +
  theme(axis.title = element_text(size = 8, vjust = +3),
        axis.text.x = element_text(size = 4, angle = 35),
        axis.text = element_text(size = 6, colour='grey20'),
        axis.ticks.x = element_blank(),
        axis.ticks.y = element_line(colour = 'black', linewidth = 0.35),
        panel.grid = element_blank(),
        panel.background = element_blank(),
        panel.border = element_rect(colour = 'grey20',
                                    linewidth = 0.5, fill = NA),
        plot.background = element_blank(),
        plot.margin = unit(c(0,0,0,0), 'cm'))

p6 <- ggdraw() +
  draw_plot(p4, x = 0, y = 0) +
  draw_plot(p5, x = 0, y = 0)

layout_f <- 'A#B'
outplot <- wrap_plots(A = p3, B = p6, design = layout_f) +
  plot_layout(widths = unit(c(6, 0.2, 6), c('cm')),
              heights = unit(c(5, 5, 5), c('cm')))
outplot

ggsave('Figure 3ab HR histograms with number of phages per article.pdf',
       width = 14, height = 6, units = c('cm'))


################################################################################
############## Distribution of host ranges across phage families ###############
################################################################################

# Select phage family-level groups with at least 15 host range data points
SHR_by_phage_groups <- phagedata %>%
  select(Phage_group, Host1species, Spotting_HR, PMID_or_Title) %>%
  drop_na() %>%
  group_by(Phage_group) %>%
  mutate(N_phages_per_family = n()) %>%
  filter(N_phages_per_family >= 15)

PHR_by_phage_groups <- phagedata %>%
```

```r
  select(Phage_group, Host1species, Plaquing_HR, PMID_or_Title) %>%
  drop_na() %>%
  group_by(Phage_group) %>%
  mutate(N_phages_per_family = n()) %>%
  filter(N_phages_per_family >= 15)

# Remove Peduoviridae, since there are not enough PHR data points
SHR_by_phage_groups <- SHR_by_phage_groups %>%
  filter(Phage_group != 'Peduoviridae')

# Assign colours to 12 most common bacterial species
SHR_by_phage_groups %>%
  ungroup() %>%
  select(Host1species) %>%
  group_by(Host1species) %>%
  count() %>%
  arrange(desc(n))

PHR_by_phage_groups %>%
  ungroup() %>%
  select(Host1species) %>%
  group_by(Host1species) %>%
  count() %>%
  arrange(desc(n))

prevalent_hosts <-
  c('Acinetobacter baumannii',
    'Escherichia coli',
    'Klebsiella pneumoniae',
    'Pseudomonas aeruginosa',
    'Pseudomonas syringae',
    'Rhizobium etli',
    'Salmonella enterica',
    'Staphylococcus aureus',
    'Streptococcus thermophilus',
    'Vibrio parahaemolyticus',
    'Yersinia enterocolitica',
    'Yersinia pseudotuberculosis')

# Based on brewer.pal(12,'Paired')
my_bacterial_colours <- c('#A6CEE3', '#1F78B4', '#B2DF8A', '#33A02C', '#FB9A99',
        '#E31A1C', '#FDBF6F', '#FF7F00', '#CAB2D6',
        '#6A3D9A', '#FFF00E','#B15928')

names(my_bacterial_colours) <- prevalent_hosts
colScale_bacterial <- scale_colour_manual(name = 'Host species',
    values = my_bacterial_colours, na.value = '#525252')

# Visualise distribution of spotting host ranges by phage family-level groups
plot1 <- ggplot(SHR_by_phage_groups, aes(reorder(Phage_group, Spotting_HR,
                                                 FUN = median), Spotting_HR)) +
  geom_boxplot(fill = NA, outlier.shape = NA, linewidth = 0.25) +
  scale_y_continuous(expand = c(0, 0.05), breaks = c(0, 0.25, 0.5, 0.75, 1),
               limits = c(0, 1.1)) +
  geom_jitter(size = 1.7, shape = 18, aes(colour = Host1species),
              width = 0.15, alpha = 0.9) +
  colScale_bacterial +
  ylab('Spotting host range') +
  theme_minimal() +
  theme(axis.title.y = element_text(size = 8),
        axis.title.x = element_blank(),
        axis.text = element_text(size = 6, colour='black'),
        axis.text.x = element_text(face = 'italic', angle = 35,
            vjust = 1, hjust = 1),
        axis.line = element_line(colour = 'black', linewidth = 0.35),
        axis.ticks = element_line(colour = 'black', linewidth = 0.35),
        legend.title = element_text(size = 8),
        legend.text = element_text(size = 6, face = 'italic'),
        legend.position = 'none',
        # Legend was drawn separately and added in Inkscape 1.3.1
```

```r
        panel.grid.major = element_blank(),
        panel.grid.minor = element_blank(),
        panel.border = element_blank(),
        panel.background = element_blank(),
        plot.background = element_blank(),
        plot.margin = unit(c(0.25,0.25,0.25,0.25), 'cm'))


# Visualise the distribution of plaquing host ranges across phage families
# Reorder phage family-level groups according to their SHR medians
PHR_by_phage_groups <- as.data.frame(PHR_by_phage_groups)
PHR_by_phage_groups$Phage_group <- factor(PHR_by_phage_groups$Phage_group,
  levels = c('Aliceevansviridae', 'Drexlerviridae', 'FLG-A', 'FLG-AS', 'FLG-G',
  'Demerecviridae', 'Straboviridae', 'Herelleviridae'))

plot2 <- ggplot(PHR_by_phage_groups, aes(Phage_group, Plaquing_HR)) +
  geom_boxplot(fill = NA, outlier.shape = NA, linewidth = 0.25) +
  scale_y_continuous(expand = c(0, 0.05), breaks = c(0, 0.25, 0.5, 0.75, 1),
                     limits = c(0, 1.1)) +
  geom_jitter(size = 1.7, shape = 18, aes(colour = Host1species),
              width = 0.15, alpha = 0.9) +
  colScale_bacterial +
  ylab('Plaquing host range') +
  theme_minimal() +
  theme(axis.title.y = element_text(size = 8),
        axis.title.x = element_blank(),
        axis.text = element_text(size = 6, colour='black'),
        axis.text.x = element_text(face = 'italic', angle = 35,
              vjust = 1, hjust = 1),
        axis.line = element_line(colour = 'black', linewidth = 0.35),
        axis.ticks = element_line(colour = 'black', linewidth = 0.35),
        legend.title = element_text(size = 8),
        legend.text = element_text(size = 6, face = 'italic'),
        legend.position = 'none',
        # Legend was drawn separately and added in Inkscape 1.3.1
        panel.grid.major = element_blank(),
        panel.grid.minor = element_blank(),
        panel.border = element_blank(),
        panel.background = element_blank(),
        plot.background = element_blank(),
        plot.margin = unit(c(0.25,0.25,0.25,0.25), 'cm'))

layout_f <- 'A#B'
HRs_by_phage_groups <- wrap_plots(A = plot1, B = plot2, design = layout_f) +
  plot_layout(widths = unit(c(6, 0, 6), c('cm')))

HRs_by_phage_groups

ggsave('Figure 5ab HRs by phage groups.pdf', width = 15,
       height = 8, units = c('cm'))


################################################################################
############ Distribution of phage host ranges by bacterial species ############
################################################################################

# Select bacterial species with at least 15 host range data points
SHR_by_bacterial_species <- phagedata %>%
  select(Phage_group, Host1species, Spotting_HR, PMID_or_Title) %>%
  drop_na(Spotting_HR) %>%
  group_by(Host1species) %>%
  mutate(N_hrs_per_species = n()) %>%
  filter(N_hrs_per_species >= 15)
PHR_by_bacterial_species <- phagedata %>%
  select(Phage_group, Host1species, Plaquing_HR, PMID_or_Title) %>%
  drop_na(Plaquing_HR) %>%
  group_by(Host1species) %>%
  mutate(N_hrs_per_species = n()) %>%
  filter(N_hrs_per_species >= 15)
```

```
# Keep bacteria with both types of bacteriophage host range data available
SHR_by_bacterial_species %>%
  select(Spotting_HR) %>%
  group_by(Host1species) %>%
  nest()
PHR_by_bacterial_species %>%
  select(Plaquing_HR) %>%
  group_by(Host1species) %>%
  nest()
selected_bacteria <-
c('Acinetobacter baumannii',
'Escherichia coli',
'Klebsiella pneumoniae',
'Pseudomonas aeruginosa',
'Salmonella enterica',
'Staphylococcus aureus',
'Streptococcus thermophilus')
SHR_by_bacterial_species <- SHR_by_bacterial_species %>%
  filter(Host1species %in% selected_bacteria)
PHR_by_bacterial_species <- PHR_by_bacterial_species %>%
  filter(Host1species %in% selected_bacteria)

# Assign colours to 8 most common phage groups
prevalent_phages <- # Phage selection is inherited from taxonomic analysis
c('Herelleviridae',
'FLG-A',
'FLG-G',
'Straboviridae',
'FLG-AS',
'Demerecviridae',
'Aliceevansviridae',
'Drexlerviridae')
my_viral_colours <- c('#66A61E', '#D95F02', '#A6761D', '#7570B3',
                      '#E6AB02', '#E7298A', '#3A5FCD', '#1B9E77')
names(my_viral_colours) <- prevalent_phages
colScale_viral <- scale_colour_manual(name = 'Bacteriophage group',
  values = my_viral_colours, na.value = '#525252')

# Visualise the distribution of spotting host ranges across phage families
plot3 <- ggplot(SHR_by_bacterial_species, aes(reorder(Host1species,
                  Spotting_HR, FUN = median), Spotting_HR)) +
  geom_boxplot(fill = NA, outlier.shape = NA, linewidth = 0.25) +
  scale_y_continuous(expand = c(0, 0.05), breaks = c(0, 0.25, 0.5, 0.75, 1),
                     limits = c(0, 1.1)) +
  geom_jitter(size = 1.2, aes(colour = Phage_group), width = 0.15, alpha = 0.9) +
  colScale_viral +
  ylab('Spotting host range') +
  theme_minimal() +
  theme(axis.title.y = element_text(size = 8),
        axis.title.x = element_blank(),
        axis.text = element_text(size = 6, colour='black'),
        axis.text.x = element_text(face = 'italic', angle = 35,
                                   vjust = 1, hjust = 1),
        axis.line = element_line(colour = 'black', linewidth = 0.35),
        axis.ticks = element_line(colour = 'black', linewidth = 0.35),
        legend.title = element_text(size = 8),
        legend.text = element_text(size = 6, face = 'italic'),
        legend.position = 'none',
        # Legend was drawn separately and added in Inkscape 1.3.1
        panel.grid.major = element_blank(),
        panel.grid.minor = element_blank(),
        panel.border = element_blank(),
        panel.background = element_blank(),
        plot.background = element_blank(),
        plot.margin = unit(c(0.25,0.25,0.25,0.25), 'cm'))

# Visualise the distribution of plaquing host ranges across bacterial species
# Reorder phage family-level groups according to their SHR medians
PHR_by_bacterial_species <- as.data.frame(PHR_by_bacterial_species)
PHR_by_bacterial_species$Host1species <-
```

```r
  factor(PHR_by_bacterial_species$Host1species,
   levels = c('Streptococcus thermophilus', 'Acinetobacter baumannii',
    'Escherichia coli', 'Klebsiella pneumoniae', 'Salmonella enterica',
    'Pseudomonas aeruginosa', 'Staphylococcus aureus'))

plot4 <- ggplot(PHR_by_bacterial_species, aes(Host1species, Plaquing_HR)) +
  geom_boxplot(fill = NA, outlier.shape = NA, linewidth = 0.25) +
  scale_y_continuous(expand = c(0, 0.05), breaks = c(0, 0.25, 0.5, 0.75, 1),
                     limits = c(0, 1.1)) +
  geom_jitter(size = 1.2, aes(colour = Phage_group),
              width = 0.15, alpha = 0.9) +
  colScale_viral +
  ylab('Plaquing host range') +
  theme_minimal() +
  theme(axis.title.y = element_text(size = 8),
        axis.title.x = element_blank(),
        axis.text = element_text(size = 6, colour='black'),
        axis.text.x = element_text(face = 'italic', angle = 35,
                                   vjust = 1, hjust = 1),
        axis.line = element_line(colour = 'black', linewidth = 0.35),
        axis.ticks = element_line(colour = 'black', linewidth = 0.35),
        legend.title = element_text(size = 8),
        legend.text = element_text(size = 6, face = 'italic'),
        legend.position = 'none',
        # Legend was drawn separately and added in Inkscape 1.3.1
        panel.grid.major = element_blank(),
        panel.grid.minor = element_blank(),
        panel.border = element_blank(),
        panel.background = element_blank(),
        plot.background = element_blank(),
        plot.margin = unit(c(0.25,0.25,0.25,0.25), 'cm'))

layout_f <- 'A#B'
HRs_by_bacterial_species <- wrap_plots(A = plot3, B = plot4,
                                       design = layout_f) +
  plot_layout(widths = unit(c(6, 0, 6), c('cm')))

HRs_by_bacterial_species

ggsave('Figure 6ab HRs by bacterial species.pdf', width = 15,
       height = 8, units = c('cm'))


################################################################################
############### Median HR values and number of literature sources ##############
################################################################################

SHR_medians_by_phages <- phagedata %>%
  select(Spotting_HR, Phage_group, Phage_genome, PMID_or_Title) %>%
  drop_na() %>%
  group_by(Phage_group) %>%
  mutate(nls = length(unique(PMID_or_Title))) %>%
  mutate(n = length(unique(Phage_genome))) %>%
  mutate(SHR_median = median(Spotting_HR)) %>%
  select(Phage_group, SHR_median, n, nls) %>%
  distinct(Phage_group, SHR_median, n, nls, .keep_all = TRUE) %>%
  filter(n >= 15) %>%
  print()

PHR_medians_by_phages <- phagedata %>%
  select(Plaquing_HR, Phage_group, Phage_genome, PMID_or_Title) %>%
  drop_na() %>%
  group_by(Phage_group) %>%
  mutate(nls = length(unique(PMID_or_Title))) %>%
  mutate(n = length(unique(Phage_genome))) %>%
  mutate(PHR_median = median(Plaquing_HR)) %>%
  select(Phage_group, PHR_median, n, nls) %>%
  distinct(Phage_group, PHR_median, n, nls, .keep_all = TRUE) %>%
  filter(n >= 15) %>%
  print()
```

```
SHR_medians_by_bacteria <- phagedata %>%
  select(Spotting_HR, Host1species, Phage_genome, PMID_or_Title) %>%
  drop_na() %>%
  group_by(Host1species) %>%
  mutate(nls = length(unique(PMID_or_Title))) %>%
  mutate(n = length(unique(Phage_genome))) %>%
  mutate(SHR_median = median(Spotting_HR)) %>%
  select(Host1species, SHR_median, n, nls) %>%
  distinct(Host1species, SHR_median, n, nls, .keep_all = TRUE) %>%
  filter(n >= 15) %>%
  print()

PHR_medians_by_bacteria <- phagedata %>%
  select(Plaquing_HR, Host1species, Phage_genome, PMID_or_Title) %>%
  drop_na() %>%
  group_by(Host1species) %>%
  mutate(nls = length(unique(PMID_or_Title))) %>%
  mutate(n = length(unique(Phage_genome))) %>%
  mutate(PHR_median = median(Plaquing_HR)) %>%
  select(Host1species, PHR_median, n, nls) %>%
  distinct(Host1species, PHR_median, n, nls, .keep_all = TRUE) %>%
  filter(n >= 15) %>%
  print()


################################################################################
############## Pairwise Mann-Whitney U tests with host range data ##############
################################################################################

# The function allows performing pairwise tests in a batch
Mann_Whitney_test <- function(Mann_Whitney_data) {
  MWt_results <- data.frame(matrix(ncol = length(Mann_Whitney_data[,1]),
                                   nrow = length(Mann_Whitney_data[,1])))
  cnames <- unlist(Mann_Whitney_data[,1], use.names = FALSE)
  rnames <- unlist(Mann_Whitney_data[,1], use.names = FALSE)
  colnames(MWt_results) <- cnames
  rownames(MWt_results) <- rnames
  column_n = 1
  repeat {
    if (column_n == length(Mann_Whitney_data[,1])+1) {break}
    a <- unlist(Mann_Whitney_data[column_n, 2], use.names = FALSE)
    row_n = 1
    repeat {
      if (row_n == length(Mann_Whitney_data[,1])+1) {break}
      b <- unlist(Mann_Whitney_data[row_n, 2], use.names = FALSE)
      MWt_results[row_n,column_n] <-
        as.numeric(as.matrix(unlist(wilcox.test(a, b))))[2]
      row_n = row_n + 1
    }
    column_n = column_n + 1
  }
  MWt_results
}

################################################################################
# Prepare data for the tests

# Order of taxa to appear on plots
flg_order <- c('Aliceevansviridae', 'Drexlerviridae', 'FLG-A', 'FLG-AS', 'FLG-G',
               'Demerecviridae', 'Straboviridae', 'Herelleviridae')
bacsp_order <- c('Streptococcus thermophilus', 'Acinetobacter baumannii',
            'Escherichia coli', 'Klebsiella pneumoniae', 'Salmonella enterica',
            'Pseudomonas aeruginosa', 'Staphylococcus aureus')

SHR_by_phage_groups <- phagedata %>%
  select(Phage_group, Host1species, Spotting_HR) %>%
  drop_na() %>%
  group_by(Phage_group) %>%
  mutate(N_phages_per_family = n()) %>%
```

```
  filter(N_phages_per_family >= 15)
SHR_by_phage_groups <- SHR_by_phage_groups %>%
  filter(Phage_group != 'Peduoviridae')
MW_SHR_data_by_phages <- SHR_by_phage_groups %>%
  select(Spotting_HR) %>%
  group_by(Phage_group) %>%
  nest()
MW_SHR_data_by_phages <- as.data.frame(MW_SHR_data_by_phages)
MW_SHR_data_by_phages <- MW_SHR_data_by_phages[match(flg_order,
                                  MW_SHR_data_by_phages$Phage_group),]

PHR_by_phage_groups <- phagedata %>%
  select(Phage_group, Host1species, Plaquing_HR) %>%
  drop_na() %>%
  group_by(Phage_group) %>%
  mutate(N_phages_per_family = n()) %>%
  filter(N_phages_per_family >= 15)
MW_PHR_data_by_phages <- PHR_by_phage_groups %>%
  select(Plaquing_HR) %>%
  group_by(Phage_group) %>%
  nest()
MW_PHR_data_by_phages <- as.data.frame(MW_PHR_data_by_phages)
MW_PHR_data_by_phages <- MW_PHR_data_by_phages[match(flg_order,
                                  MW_PHR_data_by_phages$Phage_group),]

SHR_by_bacterial_species <- phagedata %>%
  select(Phage_group, Host1species, Spotting_HR) %>%
  drop_na(Spotting_HR) %>%
  group_by(Host1species) %>%
  mutate(N_hrs_per_species = n()) %>%
  filter(N_hrs_per_species >= 15)
SHR_by_bacterial_species <- SHR_by_bacterial_species %>%
  filter(Host1species %in% selected_bacteria)
MW_SHR_data_by_bacteria <- SHR_by_bacterial_species %>%
  select(Spotting_HR) %>%
  group_by(Host1species) %>%
  nest()
MW_SHR_data_by_bacteria <- as.data.frame(MW_SHR_data_by_bacteria)
MW_SHR_data_by_bacteria <- MW_SHR_data_by_bacteria[match(bacsp_order,
                              MW_SHR_data_by_bacteria$Host1species),]

PHR_by_bacterial_species <- phagedata %>%
  select(Phage_group, Host1species, Plaquing_HR) %>%
  drop_na(Plaquing_HR) %>%
  group_by(Host1species) %>%
  mutate(N_hrs_per_species = n()) %>%
  filter(N_hrs_per_species >= 15)
PHR_by_bacterial_species <- PHR_by_bacterial_species %>%
  filter(Host1species %in% selected_bacteria)
MW_PHR_data_by_bacteria <- PHR_by_bacterial_species %>%
  select(Plaquing_HR) %>%
  group_by(Host1species) %>%
  nest()
MW_PHR_data_by_bacteria <- as.data.frame(MW_PHR_data_by_bacteria)
MW_PHR_data_by_bacteria <- MW_PHR_data_by_bacteria[match(bacsp_order,
                              MW_PHR_data_by_bacteria$Host1species),]


################################################################################
# Perform Mann-Whitney U tests

pvmx_SHR <- as.matrix(Mann_Whitney_test(MW_SHR_data_by_phages))
pvmx_PHR <- as.matrix(Mann_Whitney_test(MW_PHR_data_by_phages))
pvmx_SHR_b <- as.matrix(Mann_Whitney_test(MW_SHR_data_by_bacteria))
pvmx_PHR_b <- as.matrix(Mann_Whitney_test(MW_PHR_data_by_bacteria))


################################################################################
# Visualise the results of Mann-Whitney U tests
```

```
# Set the colour scheme
colScale_statistics <- scale_fill_manual(name = 'Mann-Whitney\nU test',
    values = c('[0,0.001)' = '#6C4A3F',
    '[0.001,0.01)' = '#957468',
    '[0.01,0.05)' = '#D0C7C2',
    '[0.05,1.1)' = '#F5F2EA'),
    labels = c('P < 0.001 ***', 'P < 0.01 **', 'P < 0.05 *', 'non-significant'),
    drop = FALSE)

# Calculate the plots
pvmx_SHR[upper.tri(pvmx_SHR, diag=FALSE)] <- NA
melted_pvmx_SHR <- melt(pvmx_SHR, na.rm = TRUE)
melted_pvmx_SHR$value <- cut(melted_pvmx_SHR$value,
                             breaks = c(0,0.001,0.01,0.05,1.1), right = FALSE)

pv_plot1 <- ggplot(data = melted_pvmx_SHR, aes(Var1, Var2, value,
                                               fill = value)) +
  geom_tile(colour = 'white', alpha = 0.9, stat = 'identity') +
  colScale_statistics +
  theme_minimal()+
  theme(axis.text = element_text(size = 6, colour='grey20', face = 'italic'),
        axis.text.x = element_text(angle = 35, vjust = 1, hjust = 1),
        axis.title = element_blank(),
        legend.position = 'none') +
  coord_fixed()

pvmx_PHR[upper.tri(pvmx_PHR, diag=FALSE)] <- NA
melted_pvmx_PHR <- melt(pvmx_PHR, na.rm = TRUE)
melted_pvmx_PHR$value <- cut(melted_pvmx_PHR$value,
                             breaks = c(0,0.001,0.01,0.05,1.1), right = FALSE)

pv_plot2 <- ggplot(data = melted_pvmx_PHR,
                   aes(Var1, Var2, value, fill = value)) +
  geom_tile(colour = 'white', alpha = 0.9, stat = 'identity') +
  colScale_statistics +
  theme_minimal()+
  theme(axis.text = element_text(size = 6, colour='grey20', face = 'italic'),
        axis.text.x = element_text(angle = 35, vjust = 1, hjust = 1),
        axis.title = element_blank(),
        legend.title = element_text(size = 8),
        legend.text = element_text(size = 6),
        legend.key.size = unit(0.3, 'cm')) +
  coord_fixed()

pvmx_SHR_b[upper.tri(pvmx_SHR_b, diag=FALSE)] <- NA
melted_pvmx_SHR_b <- melt(pvmx_SHR_b, na.rm = TRUE)
melted_pvmx_SHR_b$value <- cut(melted_pvmx_SHR_b$value,
                               breaks = c(0,0.001,0.01,0.05,1.1), right = FALSE)

pv_plot3 <- ggplot(data = melted_pvmx_SHR_b, aes(Var1, Var2, value,
                                                 fill = value)) +
  geom_tile(colour = 'white', alpha = 0.9, stat = 'identity') +
  colScale_statistics +
  theme_minimal()+
  theme(axis.text = element_text(size = 6, colour='grey20', face = 'italic'),
        axis.text.x = element_text(angle = 35, vjust = 1, hjust = 1),
        axis.title = element_blank(),
        legend.position = 'none') +
  coord_fixed()


pvmx_PHR_b[upper.tri(pvmx_PHR_b, diag=FALSE)] <- NA
melted_pvmx_PHR_b <- melt(pvmx_PHR_b, na.rm = TRUE)
melted_pvmx_PHR_b$value <- cut(melted_pvmx_PHR_b$value,
                               breaks = c(0,0.001,0.01,0.05,1.1), right = FALSE)

pv_plot4 <- ggplot(data = melted_pvmx_PHR_b,
                   aes(Var1, Var2, value, fill = value)) +
  geom_tile(colour = 'white', alpha = 0.9, stat = 'identity') +
  colScale_statistics +
```

```r
  theme_minimal()+
  theme(axis.text = element_text(size = 6, colour='grey20', face = 'italic'),
        axis.text.x = element_text(angle = 35, vjust = 1, hjust = 1),
        axis.title = element_blank(),
        legend.title = element_text(size = 8),
        legend.text = element_text(size = 6),
        legend.key.size = unit(0.3, 'cm')) +
  coord_fixed()

layout_f <- 'A#B'

wrap_plots(A = pv_plot1, B = pv_plot2, design = layout_f) +
  plot_layout(widths = unit(c(3, 0, 3), c('cm')))
ggsave('Figure 5cd Mann-Whitney with HRs by phages.pdf', width = 16,
       height = 10, units = c('cm'))

wrap_plots(A = pv_plot3, B = pv_plot4, design = layout_f) +
  plot_layout(widths = unit(c(3, 0, 3), c('cm')))
ggsave('Figure 6cd Mann-Whitney with HRs by bacteria.pdf', width = 16,
       height = 10, units = c('cm'))


################################################################################
######## Differences between the two host ranges in paired observations ########
################################################################################

paired_observ <- phagedata %>%
  select(Phage_genome, Spotting_HR, Plaquing_HR, Phage_group, Host1species,
   PMID_or_Title) %>%
  group_by(Phage_genome, Phage_group, Host1species, PMID_or_Title) %>%
  summarise(across(c(Spotting_HR, Plaquing_HR), ~sum(., na.rm = TRUE))) %>%
  filter(Spotting_HR != 0) %>%
  filter(Plaquing_HR != 0) %>%
  mutate(Delta = Spotting_HR - Plaquing_HR)

# How many paired data points?
nrow(paired_observ)

# What part of them are detectable?
detectable <- paired_observ %>%
  filter(Delta > 0.1) %>%
  nrow()
detectable / nrow(paired_observ)

# What part of them are above 0.5?
conspicuous <- paired_observ %>%
  filter(Delta > 0.5) %>%
  nrow()
conspicuous / nrow(paired_observ)

# Get the whole picture of the differences and identify all-different
# and all-equal cases visually

# Keep independently confirmed data either for phage groups or bacterial species
Delta_by_phage_groups <- paired_observ %>%
  drop_na() %>%
  arrange(Phage_group) %>%
  group_by(Phage_group) %>%
  mutate(n = n_distinct(PMID_or_Title)) %>%
  filter(n >= 3) # At least three sources for each group
Delta_by_bacterial_species <- paired_observ %>%
  drop_na() %>%
  arrange(Host1species) %>%
  group_by(Host1species) %>%
  mutate(n = n_distinct(PMID_or_Title)) %>%
  filter(n >= 3) # At least three sources for each species

plotting1 <- ggplot() +
  geom_point(data = Delta_by_phage_groups,
             aes(reorder(Phage_group, Delta, FUN = mean), Delta, colour = Host1species),
```

```
            position = position_jitter(seed = 42, width = 0.15),
            alpha = 0.9, size = 1.7, shape = 18) +
  geom_hline(yintercept = 0.10, lty = 'dashed', linewidth = 0.15) +
  # 0.10 is an estimate of the error of repeated host range tests (Data S3)
  scale_y_continuous(expand = c(0, 0.05), breaks = c(0, 0.25, 0.5, 0.75),
                     limits = c(0, 0.75)) +
  colScale_bacterial +
  ylab('Delta') +
  theme_minimal() +
  theme(axis.title.y = element_text(size = 8),
        axis.title.x = element_blank(),
        axis.text = element_text(size = 6, colour='black'),
        axis.text.x = element_text(face = 'italic', angle = 35,
                                   vjust = 1, hjust = 1),
        axis.line = element_line(colour = 'black', linewidth = 0.35),
        axis.ticks = element_line(colour = 'black', linewidth = 0.35),
        legend.title = element_text(size = 8),
        legend.text = element_text(size = 6, face = 'italic'),
        legend.key = element_rect(colour = NA, fill = NA),
        legend.key.size = unit(0.3, 'cm'),
        legend.title.align = 0,
        panel.grid.major = element_blank(),
        panel.grid.minor = element_blank(),
        panel.border = element_blank(),
        panel.background = element_blank(),
        plot.background = element_blank(),
        plot.margin = unit(c(0.25,0.25,0.25,0.25), 'cm'))

plotting2 <- ggplot() +
  geom_point(data = Delta_by_bacterial_species,
            aes(reorder(Host1species, Delta, FUN = mean), Delta, colour = Phage_group),
            position = position_jitter(seed = 42, width = 0.15),
            alpha = 0.9, size = 1.2) +
  geom_hline(yintercept = 0.10, lty = 'dashed', linewidth = 0.15) +
  # 0.10 is an estimate of the error of repeated host range tests (Data S3)
  scale_y_continuous(expand = c(0, 0.05), breaks = c(0, 0.25, 0.5, 0.75),
                     limits = c(0, 0.75)) +
  colScale_viral +
  ylab('Delta') +
  theme_minimal() +
  theme(axis.title.y = element_text(size = 8),
        axis.title.x = element_blank(),
        axis.text = element_text(size = 6, colour='black'),
        axis.text.x = element_text(face = 'italic', angle = 35,
                                   vjust = 1, hjust = 1),
        axis.line = element_line(colour = 'black', linewidth = 0.35),
        axis.ticks = element_line(colour = 'black', linewidth = 0.35),
        legend.title = element_text(size = 8),
        legend.text = element_text(size = 6, face = 'italic'),
        legend.key = element_rect(colour = NA, fill = NA),
        legend.key.size = unit(0.3, 'cm'),
        legend.title.align = 0,
        panel.grid.major = element_blank(),
        panel.grid.minor = element_blank(),
        panel.border = element_blank(),
        panel.background = element_blank(),
        plot.background = element_blank(),
        plot.margin = unit(c(0.25,0.25,0.25,0.25), 'cm'))

layout_f <- 'A#B'

wrap_plots(A = plotting1, B = plotting2, design = layout_f) +
  plot_layout(widths = unit(c(4.75, 0, 3.25), c('cm')))

ggsave('Delta by taxa.pdf', width = 18, height = 7, units = c('cm'))


################################################################################
###################### Fitness cost of broad host ranges ######################
################################################################################
```

```
fitness_data <- phagedata %>%
  select(Plaquing_HR, Lysis, EOP_above_or_eq_0.1, Host1species,
         PMID_or_Title, Phage_group, PhaTYP_pred, Phage_genome) %>%
  drop_na(EOP_above_or_eq_0.1) %>%
  filter(PhaTYP_pred == 'virulent') %>%
  mutate(High_EOP = (EOP_above_or_eq_0.1/Lysis))

# Which phages have been published in the highest number of papers?
fitness_data %>%
  group_by(Host1species) %>%
  summarise(nls = length(unique(PMID_or_Title))) %>%
  arrange(desc(nls))
# Salmonella enterica, Escherichia coli, Acinetobacter baumannii,
# Pseudomonas aeruginosa and Vibrio parahaemolyticus

# Which phages have the highest number of EOP data points?
fitness_data %>%
  group_by(Host1species) %>%
  summarise(n = n()) %>%
  arrange(desc(n))
# Acinetobacter baumannii, Pseudomonas aeruginosa and Vibrio parahaemolyticus
# are not represented sufficiently to perform linear regression analysis

# How many papers with EOP data, Salmonella and Escherichia phages excluded?
fitness_data %>%
  filter(Host1species != 'Salmonella enterica') %>%
  filter(Host1species != 'Escherichia coli') %>%
  summarise(nls = length(unique(PMID_or_Title)))

# How many hosts with EOP data with Salmonella and Escherichia phages excluded?
fitness_data %>%
  filter(Host1species != 'Salmonella enterica') %>%
  filter(Host1species != 'Escherichia coli') %>%
  summarise(n = length(unique(Phage_genome)))

fitness_data$Binary_species <- ifelse(!(fitness_data$Host1species %in%
                        c('Escherichia coli', 'Salmonella enterica')),
                                   NA, fitness_data$Host1species)

fitness_data %>%
  print(n = 91)

# Set the colour palette
my_fitness_colours <- c('#FDBF6F', '#1F78B4')
names(my_fitness_colours) <- c('Salmonella enterica', 'Escherichia coli')
colScale_fitness <- scale_colour_manual(name = 'Host species',
                  values = my_fitness_colours, na.value = '#525252')

ggplot(fitness_data,
       aes(x = Plaquing_HR, y = High_EOP, colour = Binary_species)) +
  geom_point(size = 1.2, alpha = 0.9) +
  scale_x_continuous(limits = c(0, 1), expand = c(0.008, 0.008)) +
  scale_y_continuous(expand = c(0, 0.05), breaks = c(0, 0.25, 0.5, 0.75, 1),
                     limits = c(0, 1)) +
  xlab('Plaquing host range') +
  ylab('EOP >= 0.1 / EOP > 0') +
  geom_smooth(aes(x = Plaquing_HR, y = High_EOP), linewidth = 0.4,
              method = lm, level = 0.95, se = TRUE, fullrange = FALSE) +
  colScale_fitness +
  theme_minimal() +
  theme(axis.title = element_text(size = 8),
        axis.text = element_text(size = 6),
        axis.line = element_line(colour = 'black', linewidth = 0.35),
        axis.ticks = element_line(colour = 'black', linewidth = 0.35),
        legend.title = element_text(size = 8),
        legend.text = element_text(size = 6, face = 'italic'),
        legend.key.size = unit(0.3, 'cm'),
        panel.grid.major = element_blank(),
        panel.grid.minor = element_blank(),
```

```
        panel.border = element_blank(),
        panel.background = element_blank(),
        plot.background = element_blank(),
        plot.margin = unit(c(0.25,0.25,0.25,0.25), 'cm'))

ggsave('Figure 7 Fitness cost.pdf', width = 10, height = 7, units = c('cm'))


################################################################################
################### Host ranges, morphology and genome size ####################
################################################################################

colScale_morphological_scatter <-
  scale_colour_manual(name = 'Bacteriophage\nmorphology',
                      values = my_morphological_colours)

# Spotting host ranges and genome size
SHR_vs_genome <- phagedata %>%
  select(Genome_size, Spotting_HR, Morphology) %>%
  drop_na(Spotting_HR)  %>%
  mutate(Morphology_mod = replace_na(Morphology, 'not determined'))

SHR_vs_genome$Morphology_mod <- factor(SHR_vs_genome$Morphology_mod,
           levels = c('siphovirus', 'podovirus', 'myovirus', 'not determined'))

# Histogram of spotting host range breadth distribution
SHR_vs_genome_HR <- ggplot(SHR_vs_genome, aes(x = Spotting_HR)) +
  geom_histogram(binwidth = 0.02) +
  theme_light() +
  scale_x_continuous(limits = c(0, 1), expand = c(0.008, 0.008)) +
  xlab('Spotting host range') + ylab('') +
  theme(axis.title.y = element_text(size = 8, vjust = +3, colour='grey20'),
        axis.text = element_text(size = 6),
        axis.ticks = element_line(colour = 'black', linewidth = 0.55),
        axis.text.y = element_blank(),
        axis.ticks.y = element_blank(),
        panel.background = element_blank(),
        plot.margin = unit(c(0,0,0,0), 'cm'),
        panel.grid.major.y = element_blank(),
        panel.grid.minor.y = element_blank())

# Histogram of genome size distribution
SHR_vs_genome_GS <- ggplot(SHR_vs_genome, aes(x = Genome_size/1000)) +
  geom_histogram(binwidth = 0.07) +
  theme_light() +
  scale_x_continuous(trans='log2', limits = c(2^3.5, 2^8.5),
                     expand = c(0, 0), n.breaks = 6) +
  scale_y_continuous(expand = c(0, 0)) +
  xlab('Genome size (kbp)') + ylab('') +
  theme(axis.title.x = element_text(size = 8, colour='grey20'),
        axis.text = element_text(size = 6),
        axis.ticks = element_line(colour = 'black', linewidth = 0.55),
        axis.text.x = element_blank(),
        axis.ticks.x = element_blank(),
        panel.background = element_blank(),
        plot.background = element_blank(),
        plot.margin = unit(c(0,0,0,0), 'cm'),
        panel.grid.major.x = element_blank(),
        panel.grid.minor.x = element_blank())

# Scatter plot of spotting host range distribution by phage genome size
SHR_vs_genome_SP <- ggplot(SHR_vs_genome, aes(x = Spotting_HR,
                         y = Genome_size/1000)) +
  geom_point(aes(colour = Morphology_mod), size = 0.75, alpha = 0.9) +
  theme_minimal() +
  xlab('') + ylab('') +
  scale_x_continuous(limits = c(0,1), expand = c(0.008, 0.008)) +
  scale_y_continuous(trans='log2', limits = c(2^3.5, 2^8.5),
                     expand = c(0, 0), n.breaks = 6) +
  colScale_morphological_scatter +
```

```r
  theme(axis.text = element_blank(),
        axis.ticks = element_blank(),
        panel.border = element_blank(),
        legend.position = 'none',
        plot.margin = unit(c(0,0,0,0), 'cm'),
        panel.background = element_blank())

layout <-
'A#B
###
##C'

wrap_plots(A = SHR_vs_genome_GS + coord_flip() + scale_y_reverse(),
                  B = SHR_vs_genome_SP,
                  C = SHR_vs_genome_HR  + scale_y_reverse(), design = layout) +
  plot_layout(widths = unit(c(0.5, -0.57, 5), c('cm')),
              heights = unit(c(5, -0.57, 0.5), c('cm')))

ggsave('Figure 8a SHR by genome size and morphology.pdf', width = 9,
       height = 8, units = c('cm'))


# Plaquing host ranges and genome size
PHR_vs_genome <- phagedata %>%
  select(Genome_size, Plaquing_HR, Morphology) %>%
  drop_na(Plaquing_HR)  %>%
  mutate(Morphology_mod = replace_na(Morphology, 'not determined'))

PHR_vs_genome$Morphology_mod <- factor(PHR_vs_genome$Morphology_mod,
            levels = c('siphovirus', 'podovirus', 'myovirus', 'not determined'))

# Histogram of spotting host range breadth distribution
PHR_vs_genome_HR <- ggplot(PHR_vs_genome, aes(x = Plaquing_HR)) +
  geom_histogram(binwidth = 0.02) +
  theme_light() +
  scale_x_continuous(limits = c(0, 1), expand = c(0.008, 0.008)) +
  xlab('Plaquing host range') + ylab('') +
  theme(axis.title.y = element_text(size = 8, vjust = +3, colour='grey20'),
        axis.text = element_text(size = 6),
        axis.ticks = element_line(colour = 'black', linewidth = 0.55),
        axis.text.y = element_blank(),
        axis.ticks.y = element_blank(),
        panel.background = element_blank(),
        plot.margin = unit(c(0,0,0,0), 'cm'),
        panel.grid.major.y = element_blank(),
        panel.grid.minor.y = element_blank())

# Histogram of genome size distribution
PHR_vs_genome_GS <- ggplot(PHR_vs_genome, aes(x = Genome_size/1000)) +
  geom_histogram(binwidth = 0.07) +
  theme_light() +
  scale_x_continuous(trans='log2', limits = c(2^3.5, 2^8.5),
                     expand = c(0, 0), n.breaks = 6) +
  scale_y_continuous(expand = c(0, 0)) +
  xlab('Genome size (kbp)') + ylab('') +
  theme(axis.title.x = element_text(size = 8, colour='grey20'),
        axis.text = element_text(size = 6),
        axis.ticks = element_line(colour = 'black', linewidth = 0.55),
        axis.text.x = element_blank(),
        axis.ticks.x = element_blank(),
        panel.background = element_blank(),
        plot.background = element_blank(),
        plot.margin = unit(c(0,0,0,0), 'cm'),
        panel.grid.major.x = element_blank(),
        panel.grid.minor.x = element_blank())

# Scatter plot of spotting host range distribution by phage genome size
PHR_vs_genome_SP <- ggplot(PHR_vs_genome, aes(x = Plaquing_HR,
                                               y = Genome_size/1000)) +
  geom_point(aes(colour = Morphology_mod), size = 0.75, alpha = 0.9) +
```

```r
  theme_minimal() +
  xlab('') + ylab('') +
  scale_x_continuous(limits = c(0,1), expand = c(0.008, 0.008)) +
  scale_y_continuous(trans='log2', limits = c(2^3.5, 2^8.5),
                     expand = c(0, 0), n.breaks = 6) +
  colScale_morphological_scatter +
  theme(axis.text = element_blank(),
        axis.ticks = element_blank(),
        panel.border = element_blank(),
        #legend.position = 'none',
        plot.margin = unit(c(0,0,0,0), 'cm'),
        panel.background = element_blank())

wrap_plots(A = PHR_vs_genome_GS + coord_flip() + scale_y_reverse(),
           B = PHR_vs_genome_SP,
           C = PHR_vs_genome_HR  + scale_y_reverse(), design = layout) +
  plot_layout(widths = unit(c(0.5, -0.57, 5), c('cm')),
              heights = unit(c(5, -0.57, 0.5), c('cm')))

ggsave('Figure 8b PHR by genome size and morphology.pdf', width = 11,
       height = 8, units = c('cm'))


################################################################################
#################### Sources of heterogeneity in HR data #####################
################################################################################

# Identify the most studied virus-host pairs
phagedata %>%
  select(Host1species, Phage_group, Spotting_HR, PMID_or_Title) %>%
  drop_na() %>%
  group_by(Host1species, Phage_group) %>%
  summarise(nls = length(unique(PMID_or_Title))) %>%
  arrange(desc(nls)) %>%
  print(n = 15)

phagedata %>%
  select(Host1species, Phage_group, Plaquing_HR, PMID_or_Title) %>%
  drop_na() %>%
  group_by(Host1species, Phage_group) %>%
  summarise(nls = length(unique(PMID_or_Title))) %>%
  arrange(desc(nls)) %>%
  print(n = 15)

# Load FAMD packages
library(FactoMineR) # v2.11
library(factoextra) # v1.0.7

# Import data
df <- read_xlsx('Data S2.xlsx', na = 'NA')

# Sources of heterogeneity in SHR data
shr_heterogeneity <- df %>%
  select('Host typing', 'Virus-host', 'Spotting HR',
         'Culture medium', 'tRNA predictions') %>%
  drop_na()

# Visualise the correlation between quantitative and qualitative variables
# and the principal dimensions, as well as, the contribution of variables
# to the dimensions 1 and 2
shr_res.famd <- FAMD(shr_heterogeneity, graph = FALSE)
get_eigenvalue(shr_res.famd)
fviz_screeplot(shr_res.famd)

shr_variables_famd <-
fviz_famd_var(shr_res.famd, repel = TRUE, labelsize = 2) +
  theme(axis.title = element_text(size = 8),
        axis.text = element_text(size = 6),
        axis.ticks = element_line(colour = 'black', linewidth = 0.35),
        panel.border = element_blank(),
```

```
        panel.background = element_blank(),
        plot.title = element_text(size = 8),
        plot.background = element_blank(),
        plot.margin = unit(c(0.25,0.25,0.25,0.25), 'cm'))

# Sources of heterogeneity in PHR data
phr_heterogeneity <- df %>%
  select('Host typing', 'Virus-host', 'Plaquing HR',
         'Culture medium', 'tRNA predictions') %>%
  drop_na()

phr_res.famd <- FAMD(phr_heterogeneity, graph = TRUE)
get_eigenvalue(phr_res.famd)
fviz_screeplot(phr_res.famd)

phr_variables_famd <-
  fviz_famd_var(phr_res.famd, repel = TRUE, labelsize = 2) +
  theme(axis.title = element_text(size = 8),
        axis.text = element_text(size = 6),
        axis.ticks = element_line(colour = 'black', linewidth = 0.35),
        panel.border = element_blank(),
        panel.background = element_blank(),
        plot.title = element_text(size = 8),
        plot.background = element_blank(),
        plot.margin = unit(c(0.25,0.25,0.25,0.25), 'cm'))

layout_f <- 'AB'
heterogeneity_plot <- wrap_plots(A = shr_variables_famd, B = phr_variables_famd,
                      design = layout_f) +
  plot_layout(heights = unit(c(5, 5), c('cm')))

heterogeneity_plot

ggsave('Figure 9 Heterogeneity FAMD.pdf', width = 14, height = 7, units = c('cm'))
```
