# Supplementary material for: Lytic Spectra of Tailed Bacteriophages: A Systematic Review and Meta-Analysis
Source: Viruses. 2024 Dec 4;16(12):1879. doi: 10.3390/v16121879 (PMC11680127; doi:10.3390/v16121879)

Figure S1. Phylogenetic analysis placed one bacteriophage without taxonomic annotation into the family *Drexlerviridae*. Phylogenetic tree includes the genomes from the part #1 of the vContact2 network (Figure 4) and was rooted onto the genome of Vibrio phage Va_90-11-286_p16. Annotated sequence is shown in boldface. Three bacteriophages formed an edge case and were not considered in host range analysis of bacteriophage taxa (asterisks). The tree was inferred on the basis of translated protein-coding gene sequences using ViPTree webserver.

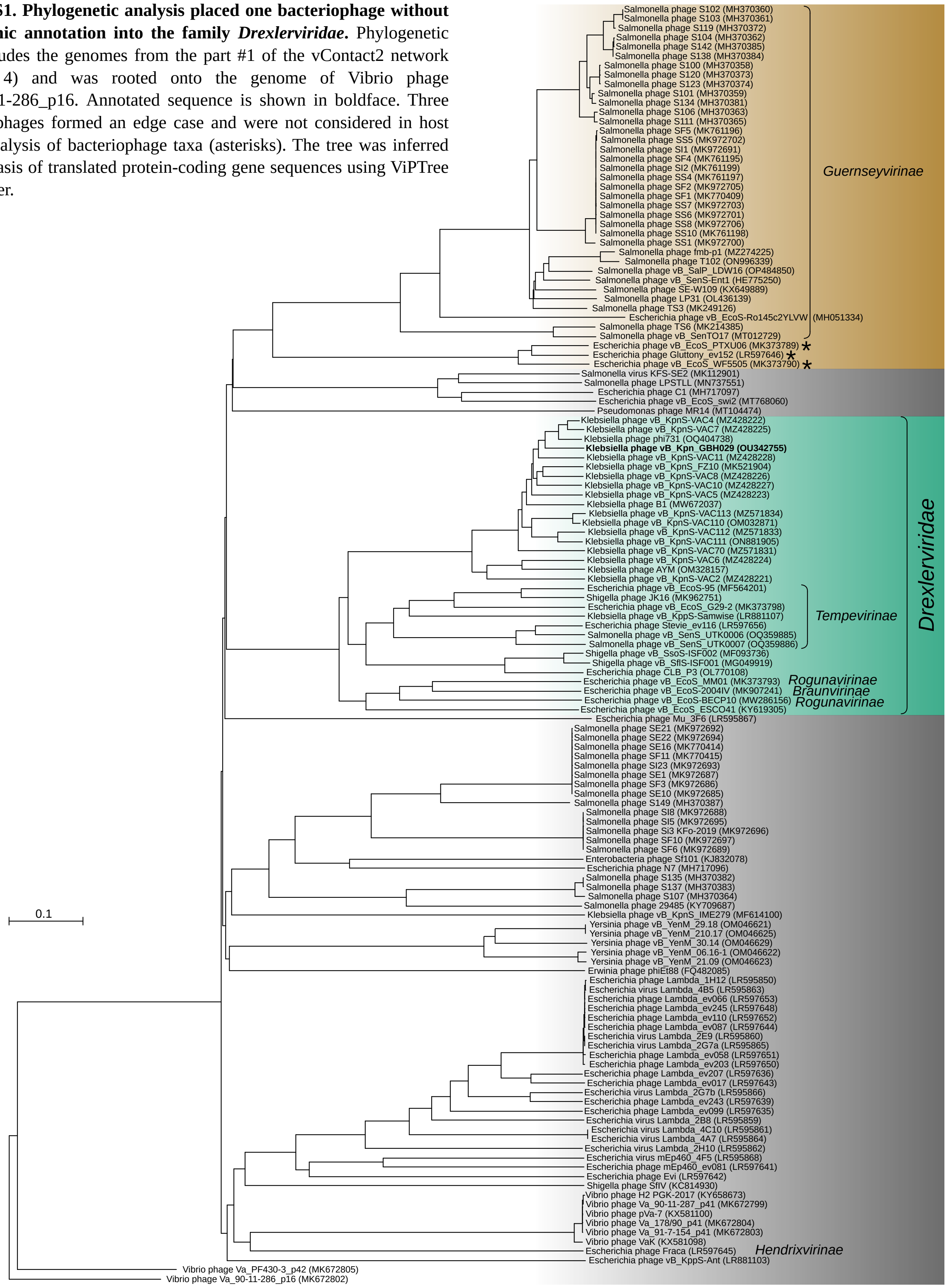

Figure S2. Phylogenetic analysis placed three bacteriophages into the family *Straboviridae*. Phylogenetic tree includes the genomes from the part #2 of the vContact2 network (Figure 4) with *Straboviridae* members and Campylobacter phage PC5. Annotated sequences are shown in boldface. The tree was inferred on the basis of translated protein-coding gene sequences using ViPTree webserver.

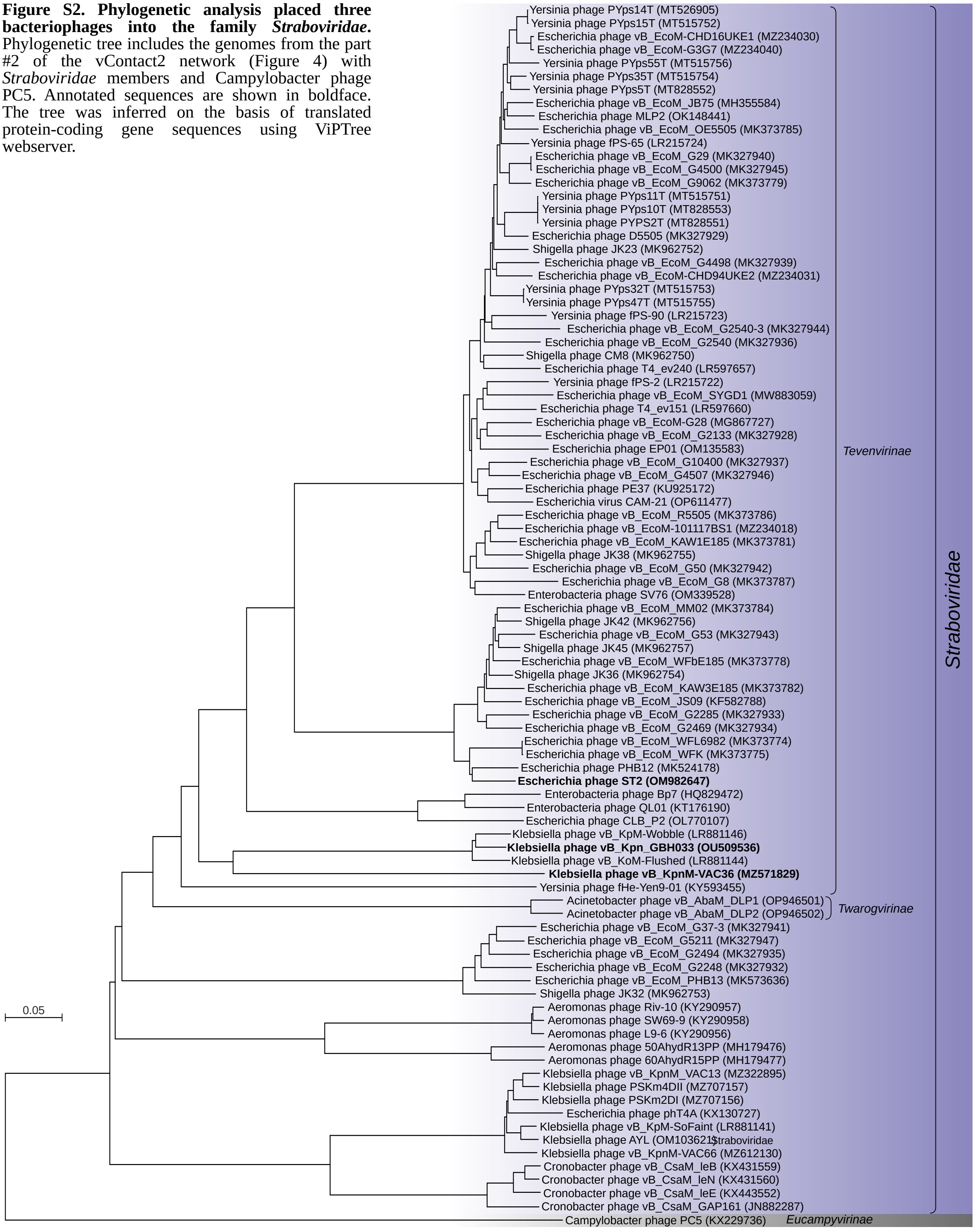

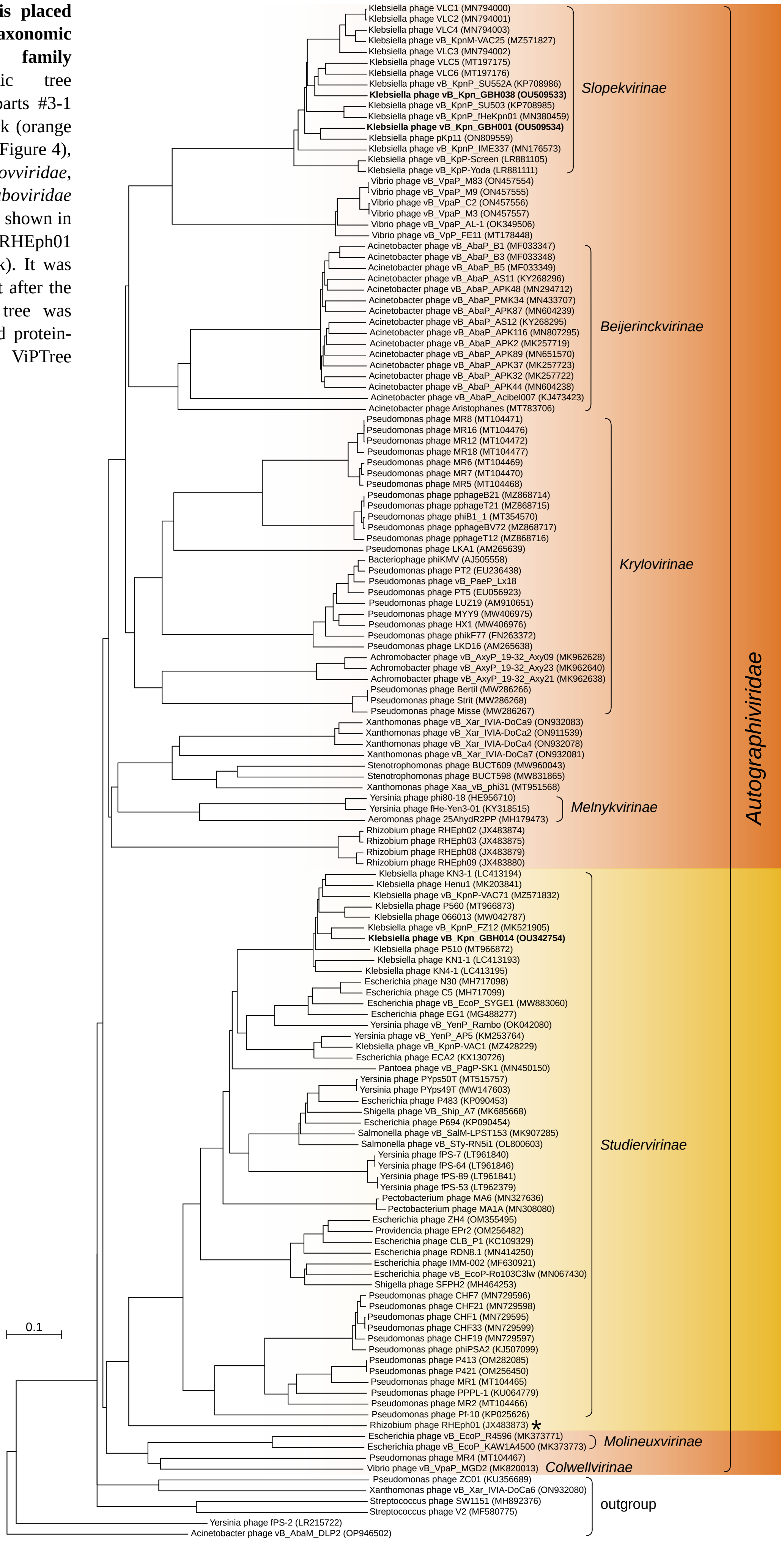

Figure S3. Phylogenetic analysis placed three bacteriophages without taxonomic annotations into the family *Autographiviridae*. Phylogenetic tree includes the genomes from the parts #3-1 and #3-2 of the vContact2 network (orange and light orange, respectively, see Figure 4), and an outgroup of *Mesyanzhinovviridae*, *Aliceevansviridae* and *Straboviridae* genomes. Annotated sequences are shown in boldface. Rhizobium phage RHEph01 represented an edge case (asterisk). It was included in *Studiervirinae* data set after the analysis of shared genes. The tree was inferred on the basis of translated protein-coding gene sequences using ViPTree webserver.

Figure S4. Phylogenetic analysis results were in agreement with GenBank annotations of *Aliceevansviridae* and related phages. The tree includes the genomes from the part #4 of the phylogenetic network (Figure 4). It was inferred on the basis of translated protein-coding gene sequences using ViPTree webserver.

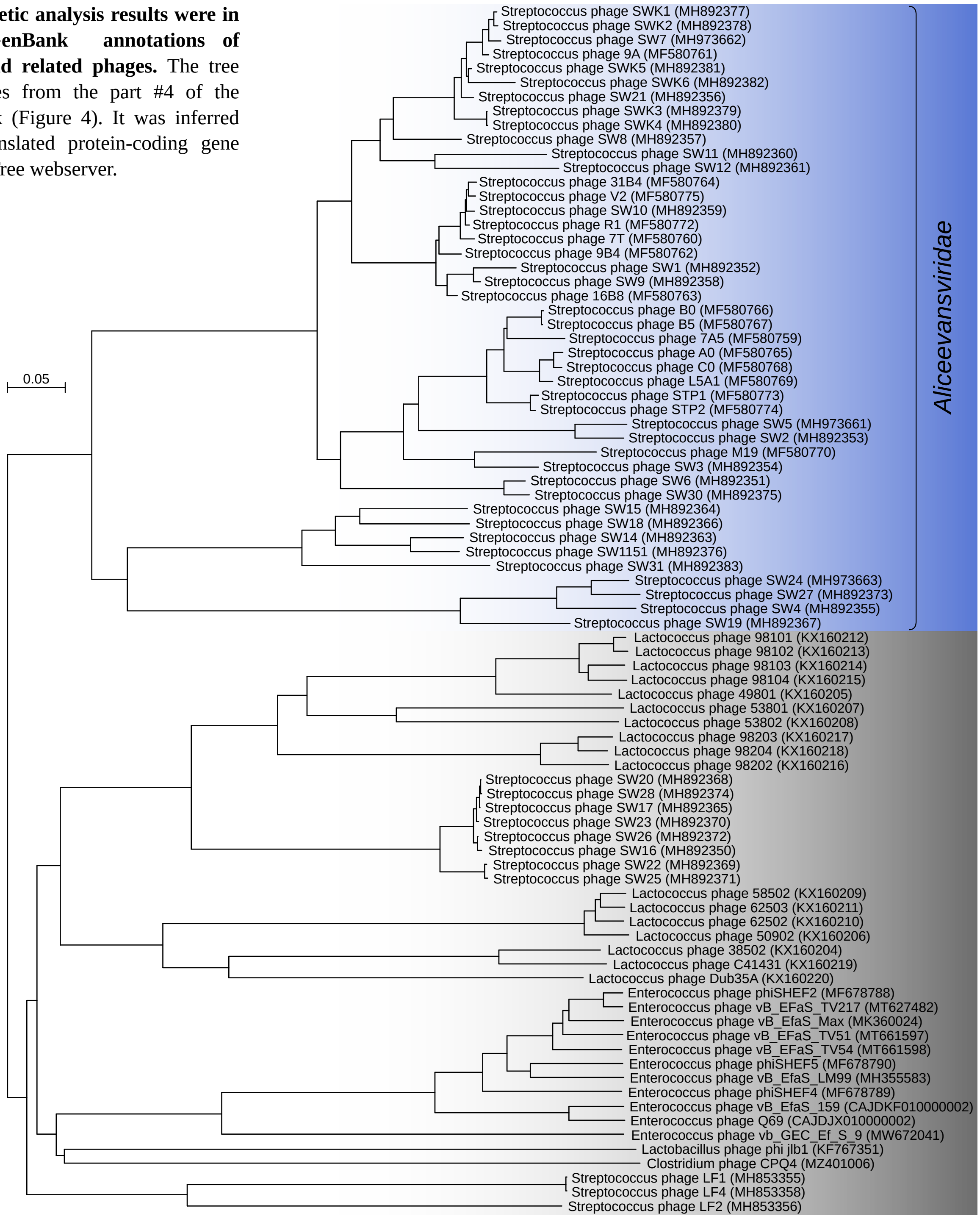

Supplement: Supplementary file 1 [file viruses-16-01879-s001.zip › Supplement/Figures S1-S4.pdf]
